# Microfabrication for Drug Delivery

**DOI:** 10.3390/ma9080646

**Published:** 2016-08-01

**Authors:** Brendan Koch, Ilaria Rubino, Fu-Shi Quan, Bongyoung Yoo, Hyo-Jick Choi

**Affiliations:** 1 Department of Chemical and Materials Engineering, University of Alberta, Edmonton, AB T6G 1H9, Canada; bmkoch@ualberta.ca (B.K.); rubino@ualberta.ca (I.R.); 2 Department of Medical Zoology, Kyung Hee University School of Medicine, Seoul 130-701, Korea; quanfs@hotmail.com; 3 Department of Materials Science and Chemical Engineering, Hanyang University, Ansan, Gyeonggi-do 426-791, Korea

**Keywords:** microfabrication, drug delivery, biocompatibility

## Abstract

This review is devoted to discussing the application of microfabrication technologies to target challenges encountered in life processes by the development of drug delivery systems. Recently, microfabrication has been largely applied to solve health and pharmaceutical science issues. In particular, fabrication methods along with compatible materials have been successfully designed to produce multifunctional, highly effective drug delivery systems. Microfabrication offers unique tools that can tackle problems in this field, such as ease of mass production with high quality control and low cost, complexity of architecture design and a broad range of materials. Presented is an overview of silicon- and polymer-based fabrication methods that are key in the production of microfabricated drug delivery systems. Moreover, the efforts focused on studying the biocompatibility of materials used in microfabrication are analyzed. Finally, this review discusses representative ways microfabrication has been employed to develop systems delivering drugs through the transdermal and oral route, and to improve drug eluting implants. Additionally, microfabricated vaccine delivery systems are presented due to the great impact they can have in obtaining a cold chain-free vaccine, with long-term stability. Microfabrication will continue to offer new, alternative solutions for the development of smart, advanced drug delivery systems.

## 1. Introduction

Nanobiotechology represents the link between engineering and life sciences [1]. On the one hand, the mechanisms of biological systems have influenced nanotechnologies, especially in their functionality and efficiency. On the other hand, engineers have used nanotechnologies to approach meaningful issues, such as health-related problems. Starting from a thorough analysis of the biological system mechanisms, the key related challenges and the final target, improved and new technologies can be developed. The successful design of fabrication methods and materials responding to the needs encountered in health science can aim to control life processes. Drug delivery, with its collection of smart systems and application of advanced materials, is a technology with such potential. 

Drug delivery systems have to face several hurdles. A first key challenge is the control of the released dose over time, which plays an essential role in medications that require a long-term therapy and/or a specific frequency of administration, as for instance insulin treatment for diabetes [2]. Additionally, resistance to a drug can be manifested by some individuals due to for example genetic defects [2] or changes in cellular processes over time [3]. The administration process is another important parameter, which closely associates with the patient’s compliance, as it is the case in intramuscular or intravenous injections [4]. Moreover, there is a need for effective delivery acting solely on the parts of the body affected by the disease [5], as the efficacy of the drug as well as the patient’s quality of life may be further compromised due to the side effects of the drug itself, such as the pain induced by chemotherapy for cancer treatment [6]. Finally, there are circumstances in which the delivery system should be able to hide the drug from the body environment until it reaches the site of interest, allowing to avoid toxicity and increase efficacy of treatment [7,8,9].

Unique advantages provided by microfabrication as compared to other techniques, such as chemistry, biology, electronics and mechanics, allow to address the issues faced by drug delivery. Microfabrication achieves fine control over the size of the devices, which has several implications when considering drug delivery. The invasiveness of the drug delivery system can be substantially reduced thanks to alternative administration methods [10]. Micro or submicron-sized devices could effectively interact at the cellular level. The drug can be used more efficiently, while lowering the toxic effects, since nano-sized devices show better uptake transcutaneously and in the pulmonary and gastro-intestinal tract [11]. In other cases, the ability to obtain larger sizes can be beneficial, for example in avoiding exhaling of the delivery system [11]. Furthermore, different components could be included on the same general platform along with the drug delivery system, such as additional drug delivery systems with diversified rates of release, sensors or electrically sensitive components [10]. Applications of the conjugation of microfabrication and drug delivery include transdermal microneedles, implants, micro and nanoparticles, which are discussed in this review.

Thus, the use of microfabrication in drug delivery applications has brought about advantages on several levels thanks to properties such as electrical sensitivity and controlled size, as well as the potential for high reproducibility of the delivery system [12,13]. For instance, implantable anode-cathode microchip reservoirs allow for controlled and triggered release [14,15]. Microelectronics can be incorporated in a pill-like sensor-reservoir device that could be administrated orally. Local delivery, preventing systemic adverse effects, is made possible through implantable devices, such as stents with embodied microneedles [16]. Microparticle carriers with added ligands achieve targeted delivery [2], as well as protection of the drug from harsh body environment conditions. These are merely a few examples of the technological enhancements that originate from the interaction of microfabrication and drug delivery.

A particular point to note with regards to microfabrication technology and nanobiotechnology is that the advances presented can be of most use to increase the global efficiency of the drug delivery system, rather than of the individual components. As an example, some of the MEMS technologies described in the following sections seek to replace intramuscular injections with different means of delivery. The products developed by these technologies may be more expensive to manufacture than a disposable syringe and needle for intramuscular injection. However, they could still be of overall lower economic cost if they remove the need of a skilled worker, such as a nurse, to administer them, or if they allow for medicines normally requiring refrigeration for transport and storage in a safe and viable state at room temperature. The elimination of pain or increasing the convenience of administration of medication can also increase patient compliance with treatment regimes, increasing their efficacy. In these ways and others that shall be discussed in more detail, improvements in delivery need to be conceived of according to how they can increase the efficiency and economy of an overall healthcare system, rather than as a simple per-unit cost basis.

## 2. Microfabrication: Technologies and Materials

In this section, the two representative fabrication technologies employed in drug delivery applications are described, namely silicon- and polymer-based fabrications. Moreover, the biocompatibility of materials frequently employed in MEMS fabrication is discussed considering their applications in humans. 

### 2.1. Microfabrication and MEMS

#### 2.1.1. Silicon-Based Fabrication

Microfabrication technology is a mature, well understood set of processes originally developed for the semiconductor industry. The minimum feature size for integrated circuitry is below 20 nm as of the publication of this work [17,18], and while the extreme measures required to achieve such high resolution are largely unnecessary for the fabrication of the devices discussed here, the methods are generally the same, and thus demonstrate the amount of room for refinement available with these methods and tools. In recent years, these processes have also been applied to generate various microstructures, often with moving components, known as microelectromechanical systems (MEMS), or if their feature size is on the nanoscale, nanoelectromechanical systems (NEMS).

Microfabrication is generally defined as a “top-down” process, in that all features are precisely defined via human action, rather than allowing chemical and physical interactions to self-assemble features with random or semi-random distribution, as in ‘bottom-up’ processes. While top-down processes have the advantage of extremely high levels of precision, they also have the disadvantage of primarily being two-dimensional in their design capacity and often requiring many serial steps to complete a design [19].

The three primary types of processes used in semiconductor and MEMS fabrication are deposition, etching, and photolithography. Deposition is the general term for applying a layer of material to another, with the most common base substrate being silicon in a semiconductor or MEMS context [20]. There is a wide variety of processes for achieving deposition, depending upon the materials being deposited and the desired final properties. A non-comprehensive list of such methods is sputtering, evaporation, electron beam evaporation, chemical vapor deposition, plasma enhanced chemical vapor deposition, electroplating, dip coating, and spin coating. These processes can be used to deposit or grow metals, ceramics, and polymers [20], and from a biological perspective are also useful in modifying the surface of materials to increase biocompatibility. For example, by depositing a layer of gold, functional groups can be bonded to the surface of a device to produce new surface activity [21].

In opposition to deposition is etching, in that it consists of removing material. Etching is material-dependent; therefore, deposition can be used to add layers of different materials to control how underlying materials are removed. While for many etching processes polymers are sufficient masking material, sometimes metals or ceramics have been used to form hard masks [22]. Here, etching processes can be broken up into two broad categories: wet and dry. Wet etches involve liquid phase, primarily aqueous, chemistry to perform chemical attacks to erode layers of material, whereas dry etching uses gas or plasma phase chemistry. Wet etching has the advantage of being generally inexpensive and exceedingly easy to perform in parallel [22].

Dry etching tends to be more expensive, since it typically requires use of vacuum chambers, purified gases stored under pressure, and high voltage power sources to generate plasma and magnetic fields, but can provide much higher etch rates and anisotropic etching [22]. While not typically used by the semiconductor industry, methods like laser or electrical ablation and physical machining can also be used to pattern on the microscale, and can be considered to fall under the same general umbrella as etching in their purpose. These processes involve high energy lasers, electrical discharges, or rapidly spinning microscale drill bits to physically remove material from a substrate, and are typically used with metals and hard polymers, with resolution generally being on the scale of hundreds of microns to tens of microns [23]. While they enable creation of more complex three-dimensional shapes, they have lower resolution than the sub-micron scale achievable with plasma and chemical etching techniques in the semiconductor industry. They are mainly employed in prototyping microfluidic devices, and creating molds and dyes for mass production of polymer structures, discussed further in the section on Polymer-based Fabrication.

Anisotropic etching is performed with an etch rate that is not equal in all directions, in contrast to isotropic etching, where the etch rate is generally the same in all directions. Isotropic etching is simple because it is how most materials respond to a chemical attack, and thus how most wet etches proceed. A significant exception is potassium hydroxide (KOH) on silicon, as the etch proceeds faster on the 100 crystallographic plane than the 111 plane, allowing for the creation of slopes of 54.7° descent from horizontal [24]. However, if strongly vertical features with high aspect ratios are desired, the dry etch processes known as deep reactive ion etches (DRIE) are needed.

DRIE consists of several forms of highly anisotropic etches that involve plasma bombardment with reactive species. While DRIE can be performed using cryogenic methods, the most common is typically some variation of the Bosch etch process [25], a dry plasma etch whereby deep vertical walls with very high aspect ratios can be achieved by a two-step process. In the first step, reactive ion plasma bombards the surface, etching away material isotropically. In the second step, the gases are changed to coat all surfaces with a fluoropolymer chemically similar to Teflon that passivates the material. When the first step is repeated, only surfaces that can be impacted by the plasma bombardment directly have the fluoropolymer etched away, while this remains on vertical surfaces and prevents etching by the reactive ion species. By alternating these two steps, vertical sidewalls are produced with a characteristic scalloped profile, where the depth of the scallops is greater with increasing duration of the etch step. Notably, depending on the application such profiles may be unwanted.

**Figure 1:**
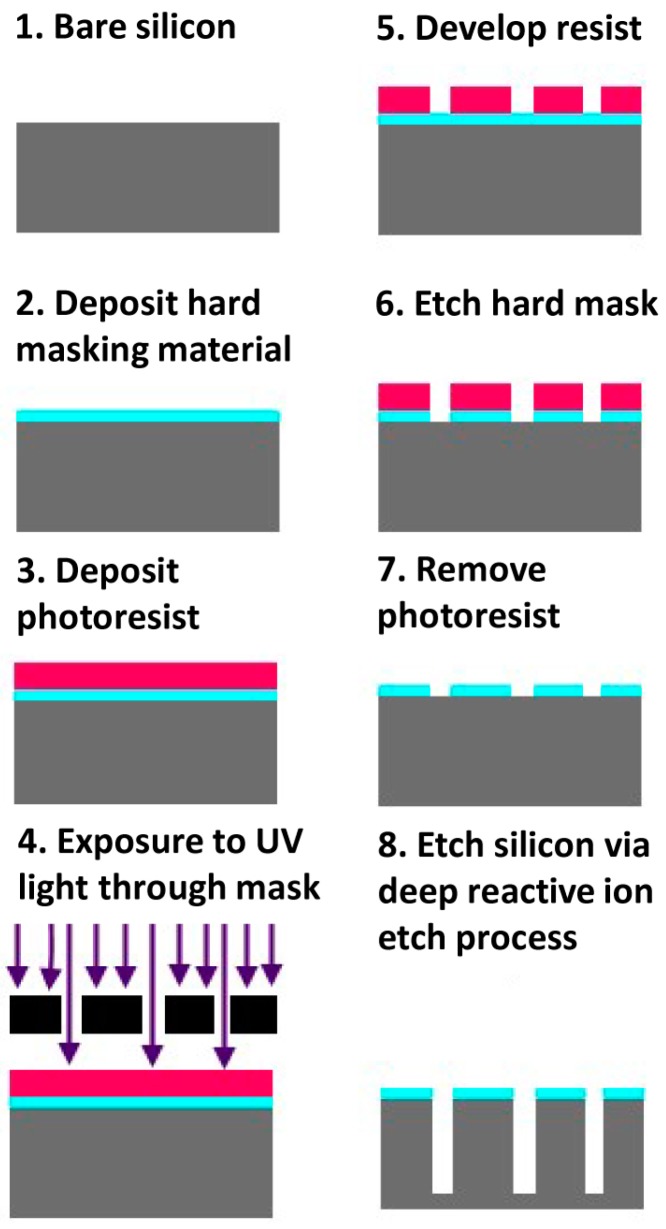
Process flow example of how a simple, single mask MEMS microfabrication process might proceed, showing deposition, photolithography, and etching.

Partially including elements from both deposition and etching, photolithography (from Greek for “stone writing with light”) is a set of processes whereby material is deposited and then selectively removed to form masks to block deposition or etching of the underlying material. As shown in Figure 1, the first step is the application of a photosensitive polymer, which can be positive or negative tone. When positive tone polymers are exposed to light of sufficient energy (typically UV light), they are more easily removed with a developing chemical. Conversely, following exposure to UV light, negative tone resists are not soluble in the developing chemical, usually due to crosslinking. Exposure to the light is controlled through photomasks, which selectively block light. Notably, transfer of the pattern from the mask to the photoresist is a two dimensional process. Therefore, it is one of the primary factors restricting top-down microfabrication to mostly planar geometries.

As an alternative to photolithography, electron beam lithography is also possible. Because of the much smaller wavelength of an electron beam, designs of much higher resolution can be achieved, at the cost of a significantly more time-consuming process compared to photolithography [26]. Whereas a single photomask can be used to repeatedly pattern an entire wafer with a single application, electron beam lithography has to perform the same rastering process, potentially taking hours, each time the design is transferred from a computer design file to the photoresist. For this reason, electron beam lithography is typically restricted to research purposes, rather than commercial applications. Another alternative is soft lithography, which will be discussed as a part of the section on polymers microfabrication.

Through repeated applications of photolithography to control etching and deposition, systems such as modern processors with billions of semiconductor gates and interconnects are produced. MEMS devices can be complex, three-dimensional systems featuring electromechanical actuators, acceleration sensors, or can be simple as channels for microfluidics. Of particular interest in the field of MEMS design is the photoresist SU-8 and the process of micromolding.

#### 2.1.2. Polymer-Based Fabrication

Elastic flexibility, biocompatibility or the ability to retain active chemicals in the molecular matrix for appropriate later diffusion are few examples of the useful properties of polymers. Among other methods of categorization, they can be divided into two primary groups: thermoset and thermoplastic polymers. Thermoset polymers undergo irreversible changes, typically crosslinking of polymer chains, following exposure to heat, light, or chemical agents. Conversely, thermoplastic polymers can be heated above their melting point, molded in their fluid state, and cooled back to solids.

SU-8 is a UV curing epoxy that serves as a negative photoresist. It is particularly useful because it can form films over 100 µm thick [27], and once fully developed, the polymer is chemically and structurally robust, allowing for structures to be made directly out of SU-8, rather than requiring further etching steps. Furthermore, since it can be obtained with a relatively high thickness and it is a negative resist, by careful use of focus and diffraction the regions of sufficient UV exposure can be controlled in three dimensions, allowing for single-step processes to create three-dimensional structures more complex than vertical pillars or channels.

**Figure 2:**
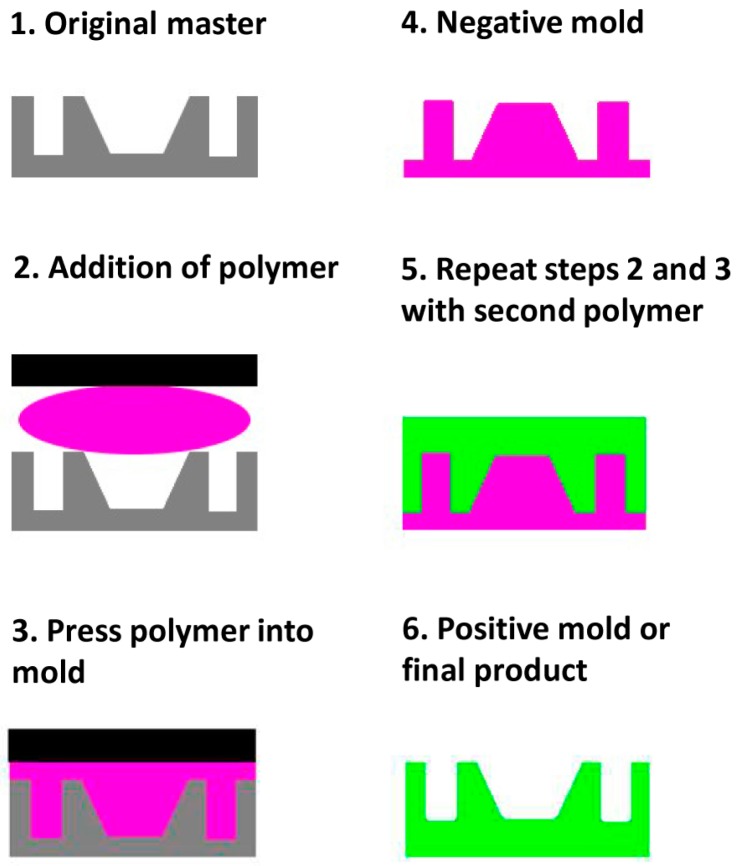
Example process flow of a molding process showing original, negative, and positive molds.

Micromolding involves producing a structure using other techniques and then applying polymers to create a negative replica (Figure 2). Depending on the design, this negative can be the final product or can serve as a mold to obtain a positive cast replica. If the original mold is delicate and difficult to prepare, or material compatibility issues arise, the positive replica can in turn serve as a new negative. However, the number of molding steps should be minimized due to accumulation of replication errors.

Other molding techniques that are applicable on the microscale and generally used with thermoplastics are injection molding and embossing. Injection molding involves forcing a thermoplastic in its liquid state into a dye mold composed of two parts that can be separated once set. Through the use of sprues and runners that can be removed after molding, complex three-dimensional shapes can be achieved. However, issues can arise at the microscale regarding the capillary pressure that needs to be overcome to achieve fine details, since the polymer can begin to set before all fine structures are filled [28]. Embossing involves using a metal dye pressing into a polymer substrate and imprinting the features of the dye into the material. This technique is capable of replicating nanoscale features in the polymer [29]. 

Since obtaining polymeric microstructures is commonly difficult from the solid state, this technique is particularly important for polymers. A main drawback of casting microstructures from molds is low three-dimensional definition. However, for certain applications [30], designs with three-dimensional complexity can be created by employing multiple fabrication steps.

Polydimethylsiloxane (PDMS) is another thermoset polymer important to the MEMS industry and to nanobiotechnology. Once set, PDMS is able to reliably retain nanometer feature sizes, and it can be used as part of a process known as soft lithography. By molding the PDMS in a master, a stamp can be formed whereby, when “inked” with a monolayer of molecules, only the highest portions of the stamp will be in contact with other surfaces. This way, micro and nanometer mask structures can be mass transferred similarly to photolithography. Importantly for nanobiotechnology, surface modification or marker molecules can be patterned on the nanoscale [31], obtaining for instance micropatterning of proteins [32,33], allowing for fine definition of zones of binding or reactivity.

Also of utility for polymer micromachining is stereolithography, which is an additive process whereby layers of liquid prepolymer are successively exposed via a focused light source, frequently a laser or UV light, to promote crosslinking and thus solidification. This allows layers to be built up into three-dimensional structures. In recent years, 3D-printers have also started to demonstrate resolutions fine enough to begin performing microfabrication work via direct printing of structures [34].

### 2.2. Biocompatibility of Materials Used in Microfabrication

While microfabrication provides the tools to produce structures and systems on the same scales that cells and smaller biological systems operate at, one particular question is the biocompatibility of the employed materials. The Food and Drug Administration (FDA) guidelines [35] regulate biocompatibility of medical devices, including bioMEMS, in the United States. With regard to materials and fabrication methods, FDA has adopted ISO 10993 [36], the internationally recognized standard for the biological evaluation of medical devices. Since the United States constitutes almost 50% of the value of the world total pharmaceutical market [37], and FDA can be highly influential in countries other than the United States [38], it is crucial to consider ahead of time which aspects are considered in testing biocompatibility.

Under ISO 10993, each device is to be tested for safety as a whole, to check for adverse effects from not just the exterior surfaces, but also from leaks or leeching from the entire device, and in the intended context of its use [36]. As such, there is no accepted list of materials on their own that are approved for use in medical devices as being biocompatible. This is, among other reasons, because different parts of the body can react dissimilarly from each other. A material approved for use in an implant inserted in the space between skin and muscle might cause a dangerous inflammation if used in an implant adjacent to neural tissue, and might degrade to produce toxic byproducts if taken orally and subjected to the low pH environment of the stomach. Another aspect is that a material intended to be in the body temporarily has different safety requirements than if intended for a long-term application [39]. Thus, biocompatibility approval is device and application specific.

In light of the FDA guidelines, biocompatibility is not investigated on the basis of the effects of the materials before the device is finalized. Instead, the manufacturing process in its entirety should be completed prior to testing the materials. Thus, not only the materials, but also the microfabrication method should be biocompatible. Furthermore, the materials employed during the production process and the extent of usage have to be carefully assessed. FDA has, for instance, recalled certain hip prosthesis produced by Zimmer^®^ (Warsaw, IN, USA) in 2015, due to the high level of manufacturing residues that could cause adverse effects [40].

Among widely used materials in MEMS are silicon, silicon compounds such as silicon dioxide, silicon carbide, silicon nitride and polycrystalline silicon [41], SU-8 [42] and polymers such as PDMS [43]. It is of extreme importance to test the biocompatibility of the materials intended for use in bioMEMS. The cytotoxicity of PDMS has been extensively investigated to constitute a reference for biocompatibility of other materials [44]. Voskerician et al. [45] studied the biocompatibility of silicon, silicon dioxide, silicon nitride, gold, SU-8 by implantation within Sprague-Dawley rats. The levels of exudate and adherence of macrophages and foreign body giant cells on the surfaces of the materials showed reduced biofouling. The biocompatibility of platinum electrodes was investigated by Geninatti et al. [46] as part of an implantable controlled drug delivery membrane in silicon. Platinum was deposited with different methods onto the silicon chip and the biocompatibility was demonstrated through viability of human dermal fibroblast cells. An in vitro study showed the successful limited adherence of macrophage and fibroblast cells onto silicon and gold-patterned silicon modified with polyethylene glycol (PEG) (Lan et al. [47]). An example of successful biocompatible surface modification of a bioMEMS material is the deposition of titanium-nitride-oxide onto stainless steel coronary stents. The coated material resulted in reduced platelet and fibrinogen binding as compared to bare stainless steel stents [48]. Another study showed good integration with Human Osteosarcoma cells of silicon coated with titanium and functionalized with Arginine-Glycine-Aspartate (Milburn et al. [49]).

Even though the aforementioned examples of in vitro and in vivo studies represent the broad efforts in determining the biocompatibility of microfabricated materials, it is of interest to relate to the evaluations that employed ISO 10993, which are more limited in variety [50,51]. The first systematic attempt in this sense was made by Kotzar et al. [50], where the biocompatibility of silicon, various silicon compounds, titanium and SU-8 were tested after deposition. Cytotoxicity and implantation evaluations were carried out in accordance with ISO 10993-5 and ISO 10993-6, respectively, and the investigated materials were found to be biocompatible. Other examples where ISO 10993-5 was employed are the evaluation of the biocompatibility of polyimides used in a pressure sensor for cardiovascular application (Starr et al. [52]), and TMMF photopolymer (Tokyo Kogyo Co., Ltd. (TOK), Kawasaki, Japan) as seal for silicon microchannels (Kalkandjiev et al. [53]). In sum, the biocompatibility of materials extensively employed in bioMEMS has been determined by in vitro and in vivo studies, and continuous increasing efforts are made to confirm these results following the recommendations of ISO 10993.

**Figure 3:**
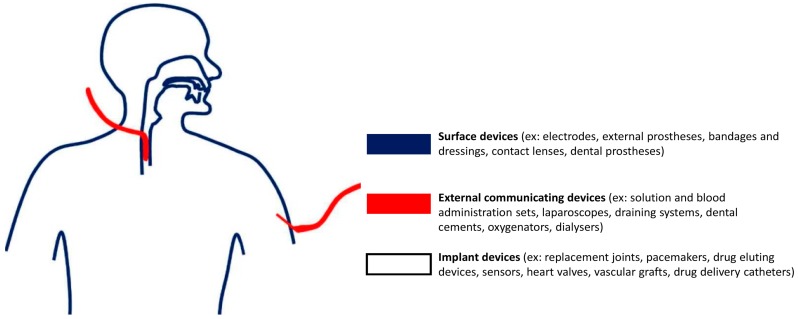
Schematic illustration of FDA classification of medical devices for testing of biocompatibility.

The selection of biocompatibility tests necessary for a material is a crucial process. Anticipating which tests a certain device should be evaluated with can be done through the matrix designed by ISO and FDA, when seeking approval for market [35,36]. Figure 3 illustrates the main classification of medical devices into surface, externally communicating and implant devices, depending on the type of contact with the human body. Cytotoxicity, sensitization and irritation tests are the common denominator for all types of medical devices. In fact, a material causing such issues can be regarded as not biocompatible without further investigation. Furthermore, a medical device can impact the body differently based on which tissues are involved in the specific application and how long for. For instance, devices solely in contact with the skin would not cause adverse systemic effects, but for devices intended for long-term use, chronic, genotoxic, implantation and carcinogenic effects would have to be tested for and not be present in the design. Moreover, devices to some extent in contact with blood pose unique risks of thrombosis, which compel the need for specific requirements of hemocompatibility.

It is worth noting that, in the case of applications involving drug release, the drug in question must also have approval for marketing. FDA regulates the approval process of new drugs under the Federal Food, Drug, and Cosmetic Act [54]. In the pre-clinical studies, the toxicity is determined through studies on animals [55]. The three subsequent phases of clinical trials determine the side effects of the drug and its efficacy by comparison with placebo treatments. If choosing to implement an already approved drug in a device, a useful reference is the Orange Book, a list of pharmaceuticals provided by FDA [56].

Finally, FDA regulations classify a product that is comprised of both device and drug (or biologic component) as a combination product [57], as for instance drug-eluting stents and transdermal patches [58,59]. Since the interaction between the two components can alter several factors of operation, such as the stability of the device and/or drug and the release profile [60], the Office of Combination products assigns primary jurisdiction to an FDA center (Center for Devices and Radiological Health (CDRH), Center for Drug Evaluation and Research (CDER), or Center for Biologics Evaluation and Research (CBER)) [61], based on the component that has the most therapeutic effect. The designated center then works in coordination with the other centers during the approval process [60].

Biocompatibility of materials, microfabrication methods and, where applicable, drugs is an essential requirement of bioMEMS. The tests that can determine such biocompatibility need to be planned ahead and in well accordance with the regulatory agencies. The evaluation of possible side effects needs to consider the entirety of aspects that affect the approval decision, such as residues, byproducts, degradation, and area and duration of contact in the body.

## 3. Microfabricated Drug Delivery Systems

To begin the discussion on how microfabricated systems can be useful for drug delivery, first one must define how the drug is to be delivered. Common routes of drug administration include the intramuscular, oral, intravenous, subcutaneous/intradermal/transdermal, intranasal, ocular, and rectal/vaginal routes. Each of these methods has its pros and cons, with the injection routes in particular having a number of problems, such as requiring skill to administer properly and safely, issues with patient compliance due to pain and fear of needles, and the proper and safe disposal of the needles after use. 

Fortunately, microfabrication allows for the creation of structures on the same scale as cells and thus for methods to bypass the body’s defenses, as for example the outer layers of the skin and cell membranes, that would not otherwise be possible at the macroscale. Additionally, working with microfabrication technologies developed for the semiconductor industry can enable direct application of integrated circuit technology in more traditional systems. Integration of computational systems can allow for the development of “smart” delivery systems that can detect changing biological conditions and thus change their responses, starting or stopping the release of medication, and obtaining optimal spatial and temporal delivery for the best therapeutic effect.

Explored here are a number of representative methods that have utilized microfabrication to improve delivery via the transdermal route, ways to improve drug eluting implants, and methods for improving drug delivery via the oral route.

### 3.1. Microneedles

#### 3.1.1. Background

The transdermal route of drug delivering involves sending the medication across the outer layer of the skin without penetrating into the deeper underlying tissues, such as the fat, muscle, or large blood vessels. Medication administered transdermally is delivered to the rest of the body via uptake by capillaries [62]. Avoiding the deeper penetration helps minimize local trauma and the activation of nerves to send pain signals. This is of considerable importance as the associated pain from routes such as intramuscular or intravenous injection can lead to needle phobias [4], which pose a problem for ensuring the best health results for people requiring continual injections, such as diabetics. These sorts of injections also require administration from skilled individuals [63], either healthcare workers or people given specific training, adding up to considerable cost for the healthcare system.

The transdermal route has been investigated for use with molecules capable of diffusing across the outer layer of the skin via gaps between cells or even transmission through cell membranes [64,65]. In certain applications, a diffusion enhancer such as dimethyl sulfoxide (DMSO) can be used to increase the uptake efficiency, but in general most medications are blocked by the outer layer of the skin. Diffusion based transdermal drug delivery also has the issue that the highest concentration of medication is received by the outer layer of the skin, where it may produce side effects. Among the transdermal medication delivery systems available on the market, patches are the most common form [66], where one representative example is the nicotine patch, used for the management of nicotine dependency for individuals wishing to quit smoking tobacco [67,68].

In contrast with diffusive methods, transdermal microneedles use a physical rather than chemical bypass, primarily by opening microscale holes in the outer layer of skin, the stratum corneum, without penetrating deeper into the tissues where pain receptors are located [69]. This allows large structures that normally could not pass through the skin, such as proteins like insulin or vaccines, to be administered transdermally. Typically, transdermal microneedles consist of an array of needles with a cross sectional radius of a few tens of microns to a few hundred microns and needle length of a few hundred to over a thousand microns. The geometry of the needles can be optimized to obtain higher skin penetration [70]. The small size of these needles is intended to produce pathways through the outer layer of skin while producing minimal discomfort to the patient. Kaushik et al. [71], demonstrated that not only application of microneedles produced significantly less pain response than hypodermic needles, but that they produced almost no pain at all. As an additional benefit, microneedle arrays require little to no training to apply properly, especially in comparison to hypodermic needles.

Transdermal microneedles can be classified by two different but interacting sets of distinctions. The first is by what materials they are made from, while the second is by how they deliver their medication. The materials used for the manufacture of microneedles must be stiff enough to not deform when pressed up against the skin and the general categories can be divided into silicon, ceramics, polymers, and metals. The methods of drug delivery available using microneedles are perforation and infusion by needle coating, dissolving needles, and hollow needles. These two sets of categorization are interrelated in that different delivery methods work best with different materials, and dissolving needles only work with materials like certain polymers.

Beginning the classification discussion, silicon is among the easier class of materials to form into microneedles directly as techniques like KOH etch can be used to form the tips and DRIE the shafts [72]. These methods are however not necessarily the most suitable for mass production, and are predominantly for prototyping purposes. These types of needles are also primarily restricted to forming solid needles for coating or for use in perforation and infusion delivery, although forming hollow microneedles with silicon is possible. One of the major uses of silicon microneedles past the prototyping stage is to serve as a template for the formation of microneedles from other materials, with the patterns being transferred to the end state material via methods such as micromolding [73].

Ceramics, including glass, are another way to form microneedles, although they tend to have fewer methods to produce very fine needles with the same regularity and repeatability as silicon. Ceramics such as alumina can be poured as a slurry into micromolds made using other techniques and then sintered solid [74]. Glass can also be formed into hollow microneedles using micropipette pulling techniques. While a well-developed set of techniques, these microneedles are also particularly fragile in comparison to the other forms as they are made of both a brittle material and are hollow. Moreover, they are more difficult to arrange into an array as they require mounting rather than being integral to the substrate.

Metal microneedles have a number of advantages over other materials in that they are strong and tough materials that hold a point well, making them good at penetrating the stratum corneum and not breaking once in the skin. Their primary difficulties are in their manufacture, requiring methods such as laser ablation [75,76], wet etching [77], and metal electroplating of template materials. Because of their general durability, these also tend to be the sorts of microneedles used for current commercial applications, such as the AdminPen™ (NanoBioSciences, LLC, Sunnyvale, CA, USA) [77] or Dermaroller^®^ (Dermaroller S.a.r.l., Friesenheim, France) [78]. The AdminPen™ is an array of metal microneedles for use as a hypodermic needle replacement, and the Dermaroller^®^ is a product intended for enhancing the uptake of skin treatment medication but presenting additional potential uses. Both will be discussed in more detail in this section.

Polymer microneedles are among the broadest categories of materials. Whereas silicon, ceramics, and metals are made of molecularly simple compounds, there is a wide number of polymers available with a variety of properties, such as mechanical strength, toxicity, ability to absorb and release materials, or solubility under physiological conditions. Thus, polymers have a wide range of applications and fabrication methods, such as direct fabrication by focusing light into cones in an SU-8 layer [79], micromolding from previously fabricated masters [80], inkjet printing [81], drawing out the polymer physically to form high aspect ratio columns [82], and stereolithography [83]. These methods can also be combined, for instance by using one method to create a polymer master to in turn generate an end product out of a polymer with more desirable properties [84], or fabricate needles in other materials, such as the previously mentioned drawing method having an end product created by electroplating the polymer in nickel to produce hollow metal microneedles [82]. The primary difficulty with polymers is that, out of all the materials, they tend to have the lowest stiffness and toughness, which can cause problems with inserting the needles or the possibility of the needles breaking off in the skin. Ultimately, the intended usage of the needles is the most crucial aspect in materials selection.

**Figure 4:**
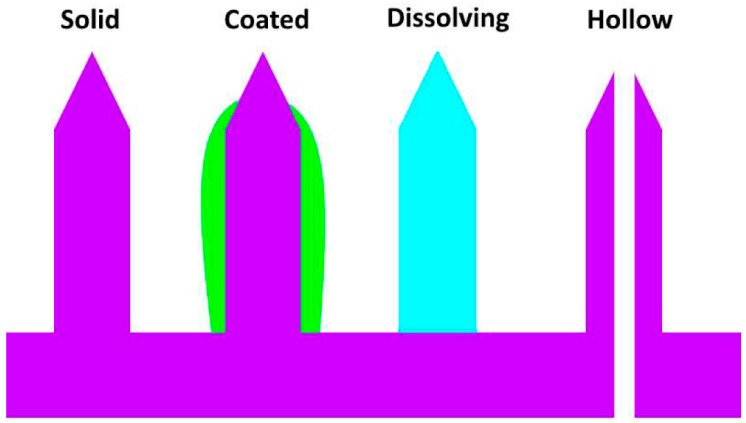
Illustration of the different kinds of microneedles; solid, coated, dissolving, and hollow.

As mentioned previously, there are four general methods of application for microneedles: perforation and infusion, coated microneedles, dissolving microneedles, and hollow microneedles (Figure 4). Perforation and infusion is the simplest method, which involves the application of a transdermal microneedle patch composed of solid microneedles. Opening of perforation channels in the skin is followed by the application of medication, diffusing through the channels, before they close, which is typically within a few hours of application of the needles [85]. The advantages of this method are that the microneedles can be made very strong to maximize penetration and minimize the chance of breaking off, and medication can be delivered for an extended period of time rather than all at once. On the other hand, the disadvantage is that the needles have the least tolerance for failure, since they must penetrate the skin and pull out successfully, and often repeatedly, such as in current commercial applications of microneedle rollers [86], without breaking. Moreover, the preferred materials for these microneedles tend to be hard and durable such as metals, and thus the consequences of a break are greater than for less durable materials, albeit still minimal in comparison to a broken traditional hypodermic needle.

Coated microneedles involve applying the medication to the exterior of the needles and having the medication dissolve for bolus delivery. The very small amount of volume that can be applied limits the medications that can be delivered by these methods to very high potency pharmaceuticals, where the low volume will not be a hindrance to efficacy [87]. A major advantage of coating the needles is that the drug is not only delivered as a bolus, but also quickly, through appropriate choice of adjuvants and excipients as part of the coating. Additionally, since the coated needles lose volume during application, retraction is facilitated by their smaller size compared to insertion.

Dissolving microneedles are exclusively made from polymers and polysaccharide materials [87] that break down under physiological conditions, releasing medication stored within their matrix or opening channels to a backing layer containing a reservoir of medication. While limited to extended release formulations, a major advantage of dissolving microneedles is the absence of a safety concern about needles breaking off inside the skin, since they are inherently biodegradable and can simply dissolve away [88]. A further positive aspect is that if the dissolvable material is designed to have different dissolution rates under different conditions at the surface of the skin—such as temperature, pH, solute content of intercellular fluid—then it has the potential for development of “smart” transdermal patches, which can self-adjust their release rates according to changes at the site of application. A major disadvantage is that the materials used are inherently weak, limiting penetration ability. As an example, buckling has been determined to be the main cause of mechanical failure in sugar microneedles [89].

Hollow microneedles function the most similarly to traditional hypodermic needles in that they provide a channel for the medication to flow through. Appealing advantages are that such needles can be used to deliver large amounts of medication quickly and in a sustained manner, and they can be used to draw samples rather than merely deliver them [72]. This allows for the possibility of a system that can sample, analyze, and alter its delivery patterns in real time. The two primary drawbacks to hollow microneedles are that they are by far the most complex to fabricate [90], and they are mechanically the weakest due to the compromises inherent in making a hollow tube versus a solid column [91].

**Figure 5:**
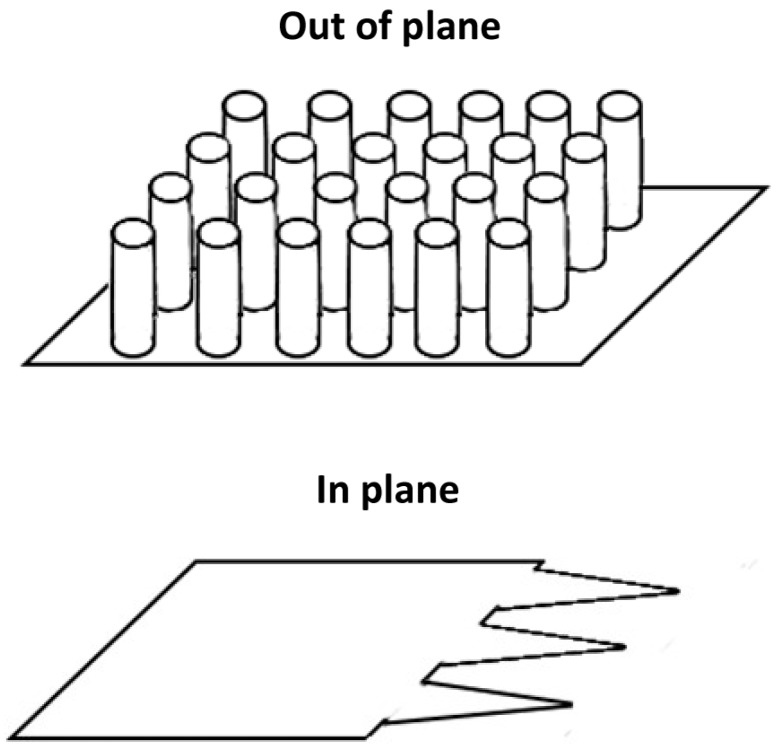
Illustration of the differences between out-of-plane and in-plane microneedle designs.

As a final distinction, while most microneedles are fabricated as out-of-plane arrays, there are a few designs that use in-plane needle structures. The difference between the two is illustrated in Figure 5: out-of-plane structures are perpendicular to the substrate surface, while the in-plane structures are machined directly out of the substrate. For out-of-plane structures, it is significantly easier to produce large arrays [92], which are particularly useful for coated and dissolving microneedle designs. The in-plane designs can have more robust mounting structures and additional structures can be patterned into them, such as microchannels [93], that can relatively easily be sealed to produce hollow microneedles. This makes the structures more useful for perforation and infusion and injection style delivery schemes.

#### 3.1.2. Status

Early research on microneedles focused on demonstrating successful fabrication of the needles, penetration of the skin, and increase of diffusivity across the skin [69]. However, as these early experiments proved that microneedles could work as envisioned, more specific applications are being pursued. While much effort has targeted applications for local delivery [94] or where slower release into the general system of the body is not an issue or even desirable—such as in the case of vaccine delivery—the utility of transdermal microneedles for drug delivery has expanded to include a larger variety of applications, such as insulin delivery [95]. This section shall review the various applications that have currently been investigated or approved for usage.

Early experiments were mostly concerned with demonstrating whether the microneedles could carry marker molecules across the skin. Use of dyes and other marker molecules continues to be a key component of investigation for new needle shape designs or fabrication techniques as they provide a useful and easily obtained baseline set of tests. Among the early tests, Henry et al. [69] used silicon microneedles fabricated via photolithography and DRIE to penetrate human skin obtained via autopsy and from plastic surgery, and then examined the change in permeability to calcein, a fluorescent dye. Their study showed that microneedles demonstrated an in vitro drop in skin resistance by 4 orders of magnitude, indicating the viability of microneedles as a concept. Another early study by Wei-Ze et al. [96] used galantamine and Evans Blues as markers for testing microneedles in comparison to a single microneedle and to skin scraping. With in vitro rat skin tests and in vivo human skin, they were able to demonstrate a noticeable improvement in permeability, while causing less damage than traditional methods of puncture. Both of these studies used solid microneedles to open channels in the skin before applying the marker chemicals, and thus fall under the perforation and infusion method.

With microneedles, the first thought for use is in applying medication to a local area, and as an example of this a pharmaceutical treatment for fecal incontinence using topically applied phenylephrine has been shown to be enhanced in rats with a microneedle perforation pretreatment [94]. The treatment that used both the medication and the microneedles increased anal pressure from the baseline of approximately 10 cmH_2_O to over 40 cmH_2_O within 20 min and it took over 4 h for the pressure to return to baseline. None of the other treatments showed any improvement over the subjects given no treatment at all, demonstrating not just the efficacy of treatment but also that transdermal microneedles can allow for treatments not normally possible. Thus, transdermal microneedles serve both as an alternative delivery method alongside more traditional routes, and to enable new forms of treatment.

Another form of treatment of local conditions that can benefit from the use of transdermal microneedles is in improving the application of local anesthetics. While lidocaine is already available in forms such as topical ointments and creams, if microneedles eliminate waste of excess application, there can be significant overall savings by using less medication for the same effect. Lidocaine also serves as a good test case for examining the functionality of microneedles beyond that of marker dyes, since it is a well understood medication that is safe at the doses generally administered, with an easily trackable effect. Most of the methods of delivery have an example using lidocaine as the delivered pharmaceutical. Transdermal patches with needles coated in lidocaine have been shown to have retention in pigs deemed sufficient to have anesthetic effects [97], and these effects can be enhanced with the addition of adjuvants [98]. Hollow microneedles used to deliver lidocaine in human subjects showed both minimal pain and the same efficacy as hypodermic injection [99]. Dissolving microneedles are also seen as an option for lidocaine delivery [100]. A structure with solid but lidocaine permeable polymer microneedles also showed promise for use in rapid and sustained delivery of lidocaine [101].

Of particular importance with microneedles is the ability to apply minute amounts of potent drugs to a precise location on the skin, reducing the impact of these extremely dose sensitive materials. One proposed usage is the improved delivery of botulinum toxin for the treatment of disorders such as focal hyperhidrosis [102]. Botulinum toxin is one of the most potent neurotoxins currently know, but it has a number of therapeutic and cosmetic applications, with the treatment of focal hyperhidrosis, a condition involving an excess production of sweat that can have significant psychological and social effects on a sufferer. By using in-plane microneedle fabrication, nanoliter sized holes could easily be made in the interior of the microneedles that could be loaded with the active ingredient and a gelling agent to hold it in place. 

Microneedles can be used to open new delivery paths for medications not only for local application, but also affecting the whole body. Use of naltrexone and naltrexol, medications intended for the treatment of addiction, tends to be hampered by the methods of delivery. The oral route has poor bioavailability, meaning that a patient is not necessarily getting the proper dosage, and low patient’s compliance [103]. Naltrexone can also be delivered by injected depot [103]. This method however has the problem that medication can only be interrupted via surgical intervention. Since the purpose of these drugs is to block opiates from functioning, if a patient undergoing treatment requires emergency surgery before the injection has run its full 30-day course, this will involve significant pain for the patient, reducing patient use of this option. Fortunately, Banks et al. [104] have shown feasibility of treatment with transdermal microneedle patch. They used a five pronged in-plane microneedle array to puncture the skin, and high permeability of the protonated drug formulation was obtained in vitro in harvested guinea pig skin.

Another non-local application of transdermal microneedles is the delivery of heparin for the treatment of deep vein thrombosis and pulmonary embolism. Heparin is a glycosaminoglycan anticoagulant, with low molecular weight heparin being preferred for clinical use due to a longer shelf life and less bleeding in comparison to other forms of heparin [105]. Despite this preference for the low molecular weight form, these are large, highly charged molecules that are poor candidates for traditional transdermal routes. This compound has been successfully loaded into dissolving microneedles for delivery to rats [105] and the therapeutic levels were maintained for hours. As an additional advantage, reducing the amount of skin penetration by an anticoagulant reduces the possibility of complications.

Because management of diabetes requires frequent injections of insulin, particular attention has been paid to making the delivery of insulin easier, safer, and less painful, which well fit the properties of delivery via microneedles. Early studies were done with Sprague Dawley rats artificially induced to have diabetes and showed the efficacy of insulin delivered via microneedle patches [95]. By applying an electric field, the diffusion of nanovesicle encapsulated insulin can be greatly enhanced via iontophoresis across microneedle penetrated guinea pig skin [106], offering another method of delivery and control. These methods can in fact help produce both a basal dosage and on demand bolus delivery dosages [107]. Dissolving microneedles composed of hyaluronic acid have been shown to safely deliver insulin to rats, and remained viable after storage for a month at a variety of temperatures between −40 °C and 40 °C, showing extremely strong potential for widespread human application [108]. The same applicability of delivery of insulin via dissolvable microneedles has been shown also in dogs [109]. An early study in humans involving five subjects shows that this technology has clinical promise beyond in vitro and in vivo studies [110].

There are currently only few commercial products related to microneedles due to the relative novelty of the designs and the long test periods that may be required for approval with human use [111]. Nonetheless, there are some products that are starting to enter into the market.

**Figure 6:**
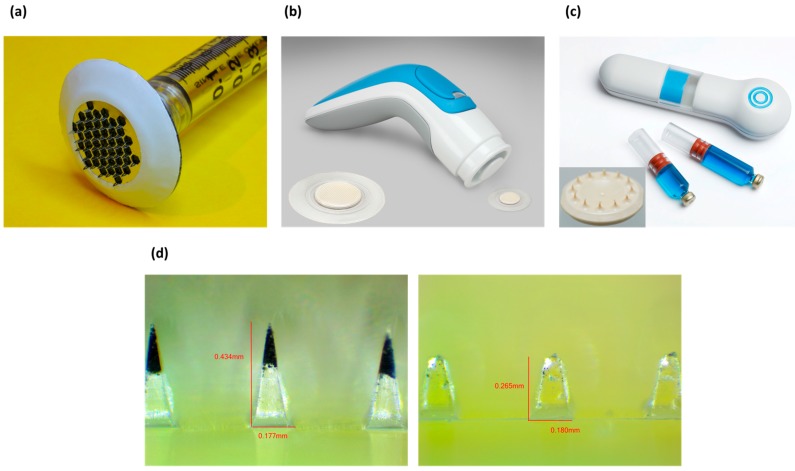
Representative examples of microneedle technology for drug delivery. (**a**) AdminPen 1200 (image provided courtesy of NanoBioSciences LLC); (**b**) 3M™ Solid and (**c**) Hollow Microstructured Transdermal System (images provided courtesy of 3M); (**d**) MPatch™ Mini dissolvable microneedles before (left) and after (right) dissolution (images provided courtesy of Micropoint Technologies Ltd., Singapore).

The AdminPen™ device is a product of the AdminMed company that features a multi-role microneedle array as the central component [77] (Figure 6a). The AdminPatch™ microneedle array is a set of metal microneedles on a hexagonal wireframe mesh that can be attached to a reservoir such as a standard syringe. While the microneedles do not provide a complete path like a fully hollow design, they still have the capacity to penetrate past the stratum corneum and provide a pathway for medication to flow through the holes produced. As an additional advantage, the use of solid tips presents reduced possibility of tissue clogging the needles during the insertion process. This design is intended as a direct replacement for hypodermic needles, providing a painless method of administration while still working with existing syringe infrastructure, and not requiring the reformulation of pharmaceuticals to be coated onto microneedles.

3M has introduced a microneedle system for drug delivery as well, with variations on the product involving both solid microneedles and hollow microneedles (Figure 6b,c). 3M systems consist of a 1 cm^2^ array of polymeric needles molded with lengths of 250, 500 or 700 µm and 1500 µm, for solid and hollow microneedles respectively [112,113]. The drug is coated and dried on top of the solid needles, while the hollow needles can deliver up to 2 mL of liquid drug formulation [114].

A platform that employs dissolving microneedles is MPatch™ Mini produced by Micropoint Technologies (Figure 6d). The microneedles are made of hyaluronic acid and can be applied to the skin by the patient using a spring-based mechnanism [115]. The drug or vaccine to be delivered is incorporated in the tips of the needles, which instantly dissolve after application (about 10 s).

Many of the current commercial microneedles products involve the perforation method, whereby the microneedles open holes in the skin to allow later application of medication to diffuse through the opened channels. The Dermaroller^®^ is one example [78], and involves the usage of a stainless steel roller studded with needles 150–1500 µm tall to perforate the skin in advance of application of dermatological medication. Since a commercial product already allowed for use by humans greatly expedites the ability to obtain approval for use and production using pre-existing facilities [111], investigating such products for other applications is an obvious direction to take. Use of these commercially available rollers has also been explored for the enhancement of transdermal insulin delivery [116]. Zhou et al. used three types of commercially available rollers with needle lengths of 250 µm, 500 µm, and 1000 µm to perforate the skin of rats to test the ability for topically applied insulin to control blood glucose levels. After initial tests with water permeability and dye delivery, it was found that by using the microneedle roller topically applied insulin could produce changes in blood glucose similar to delivery by hypodermic needles. Further research is also being done to improve rollers technology for drug delivery [86].

The Nanomed device is an array of silicon microneedles that is used for pretreatment of cosmetics and acne medication application [117], that has shown also an increase in speed of onset and degree of reduction of pain for the delivery of the topical anesthetic dyclonine [118]. Finally, the Nanopatch™ is a microneedle drug delivery system intended for use with vaccines [119] and will be discussed more thoroughly in Section 3.4.

### 3.2. Implants

#### 3.2.1. Background

Drug releasing implants are an important, developing field for improving the delivery of pharmaceuticals to a patient over an extended period of time. These have advantages in terms of user compliance with taking their medication in that they do not require repeated conscious effort, and greatly reduce burdens of pain or expertise required for operation [120,121]. This means that implants are of particular importance for people requiring treatments such as daily or more frequent injections of medication on a long term basis. The primary disadvantage is obviously that the devices require implantation, which generally will be at least a minor outpatient surgery, and will require further procedures to remove, replace or refill the pharmaceutical reservoir [122]. However, depending on the condition being treated, this could be considered an advantage, as such procedures could be done under anesthetic and thus be less painful and disruptive in comparison to the aggregate of daily maintenance procedures without the implant. Of a greater concern is that the implant itself should produce a minimal inflammatory immune response to its presence inside the patient as a foreign element [122]. Fortunately, there are decades of research into other implants and packing within biocompatible materials. Nevertheless, more complex implants may have components used for sensing physiological conditions that are exposed to the rest of the body, requiring careful consideration of materials used.

**Figure 7:**
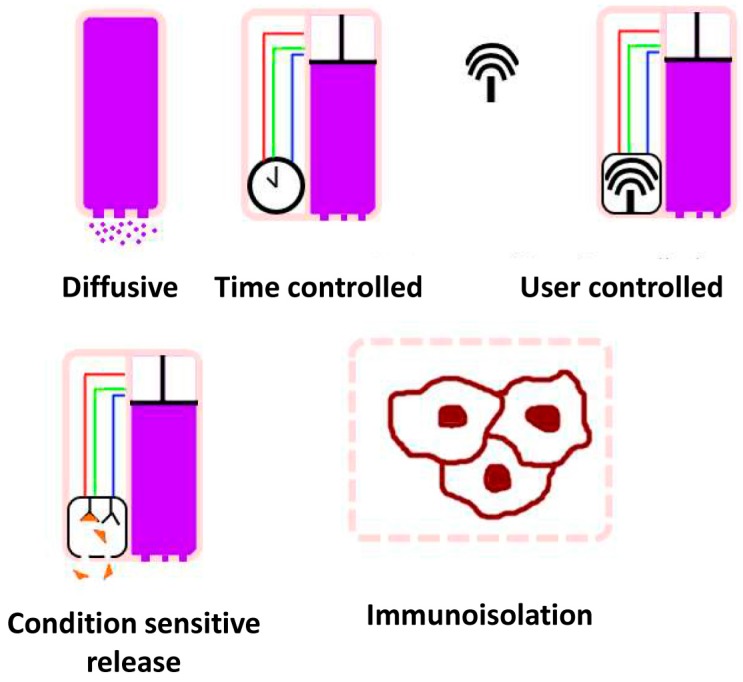
Generalized different types of implants, showing the different methods in which implants can be triggered to release their pharmaceuticals: diffusive, time controlled, user controlled, condition sensing, and immunoisolation.

Implants for drug release can generally be classified according to their method of delivery, as shown in Figure 7, which divides them into five broad categories: diffusive, time controlled, user controlled, condition sensitive, and immunoisolating. With diffusive implants, medication is released via simple diffusion from a reservoir, typically a semipermeable polymer that can retain the active materials for the expected lifespan of the implant. While very simple, the release profile is typically unresponsive to dynamic conditions, making them unsuited to many tasks. Additionally, the medication must be matched to a diffusion control material that will produce the desired results of stability before release and a suitable release profile. This matching may in fact be impossible to obtain, typically because release would be too fast or slow to achieve therapeutic results, eliminating some medications from consideration.

For time controlled release, a mechanism is used to control when medication is released into the body based on a generally timed mechanism. While theoretically this could be as complicated as a clock connected to a mechanical motor, more typical mechanisms are a seal that erodes away at a set rate to open a reservoir for release into the body. The primary advantage time controlled implants have over simple diffusive ones is that they can be loaded with medications that would not have a favorable diffusive profile—typically extremely high potency drugs given the often microscopic release volumes. While generally not responding to dynamic conditions, many of the considered designs use erosion rates to control when their reservoirs open, so that the capping materials have different erosion rates according to clinically significant conditions (e.g., a symptom involves a change in body temperature or pH of interstitial fluid in a way that such conditions will accelerate the release of the next dose). However, these effects can also work in an opposite manner, by delaying the release of a necessary dosage or bringing on an unwanted early release, and thus great care is required in material selection and method of release.

Increasing in sophistication considerably, user controlled implants will typically require at least moving and/or electronic parts. While not entirely providing the ease of use of other implants in that they cannot deliver medication independently, they often greatly lower the effort required for receiving medications, particularly for injected ones. Many patients would prefer to press a button once a day over a daily injection. These implants can also serve to reduce trauma to delicate areas such as the eyes by only puncturing one hole for the implant, rather than one hole each time a delivery of medication is required.

Condition sensitive release implants are perhaps the most ambitious design proposals, in that they would possess both a medication reservoir and a method of sensing physiological conditions relevant to the delivery of the medication to time release precisely to changing and unpredictably dynamic conditions. The archetypical example of this would be an implant for a diabetic patient that could detect blood sugar levels and release insulin as needed, functioning almost as an artificial set of pancreatic islet cells. These types of implants are by far the most complex, and as many of the sensing systems themselves are still experimental when condensed to the sizes necessary for implantation, these devices remain a theoretical design goal in most instances [123,124].

Finally, there are immunoisolating implants, which use advances in the manipulation of materials on the nanoscale to produce membranes that are permeable to simple molecules such as oxygen, carbon dioxide, and sugars, but are impermeable to larger and more complex molecules such as antibodies [125]. These implants can thus be almost seen of as a condition sensitive implant by hybridizing a diffusive implant and a traditional organ or tissue transplant.

#### 3.2.2. Status

The most common form of drug releasing implant currently in use is the contraceptive implant, with several commercially available products based around the same general idea: a set of polymer rods loaded with contraceptive medications inserted subcutaneously such that the chemicals will diffuse out independently over a period of several years, typically three to five [126,127]. Such devices serve as the archetype for the features that drug releasing implants should offer: long-term, slow release of medication, with reduced inefficacy due to patient’s non-compliance to frequency of treatment. While useful, these sorts of diffusive implants have limitations in terms of what sort of drugs can be loaded into them. There has however been a major improvement in the design of other implants using MEMS methods by incorporating drug-eluting features.

Of particular interest are anti-restenosis stents. Coronary artery disease is mainly caused by the accumulation of plaque on artery walls in a process known as coronary atherosclerosis, reducing the diameter of the artery and thus the ability of blood to flow [128]. Surgical intervention can be used to clear the blockage and widen the artery, but after surgery the diameter of the artery can narrow again via restenosis, which involves different mechanisms of narrowing from the original atherosclerosis [129]. One surgical tool is to place stents to prevent elastic rebound, but new cells can grow over the stent during restenosis, forming what is essentially a scar [130]. However, using techniques from MEMS, drugs can be loaded onto the stents to encourage the arteries to heal in a productive manner [131,132]. The structures used to load these drugs include the formation of microneedles similar to those discussed in Section 3.1 [16], which harmlessly pierce the epithelial layer of the artery wall to deliver the medications to the cells rather than to the bloodstream. These sorts of implants are particularly useful in that they can be loaded with high intensity drugs that would have adverse systematic effects if taken generally.

**Figure 8:**
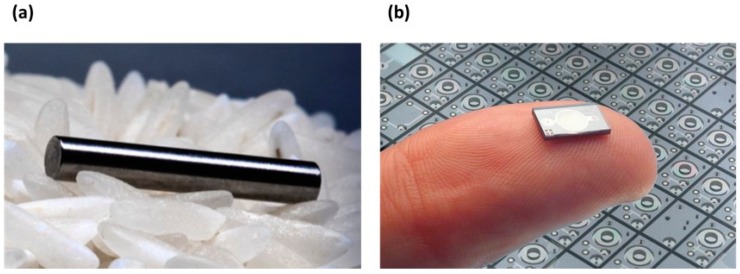
(**a**) Implantable drug reservoir with NanoPortal^TM^ membrane. The size of the device is comparable to that of a grain of rice (image provided courtesy of Nano Precision Medical, Inc., Emeryville, CA, USA); (**b**) MIP micropump for implantable drug delivery system (image provided courtesy of Debiotech S.A., Lausanne, Switzerland).

An example of a diffusive implantable drug reservoir is the technology developed by Nano Precision Medical (Emeryville, CA, USA) (Figure 8a) [133]. Their device can be small enough (2 mm) to be implanted subcutaneously by using a needle [134]. The drug is loaded in the reservoir and it passively diffuses out through the NanoPortal™ (Nano Precision Medical, Emeryville, CA, USA) membrane after implantation. The membrane consists of vertically aligned titanium nanotubes obtained by inductively-coupled plasma deep etch, and the side in contact with the body is treated with electrochemical anodization to increase biocompatibility by generating a layer of titanium oxide. Although the device does not include moving or electrical parts, a constant delivery rate can be achieved by tailoring the hole sizes of the nanotubes to the sizes of the molecules to be delivered.

As an interesting bridge between a purely diffusive delivery system and a timed implant system, there is an osmotic pressure driven implant known as the DUROS^®^ implant (ALZA, Mountain View, CA, USA) [135]. The key feature and innovation of this implant over other diffusive systems is that release is controlled not by the diffusion of the medication out of the implant, but by the diffusion of water into the implant. One side of the implant absorbs water in, causing the swelling of a polymer that physically pushes on a plunger, causing medication to be forced out of a release port. By controlling the release rate via the absorption of water, a much larger design space is made available to make an implant suitable to individual patients. So long as the medication is stable at physiological conditions for the expected period of delivery, it can be delivered, with the osmotic engine able to be tailored to the drug and patient dosage schedule. Thus, while the mechanism of actuation is based off of diffusion, the actual effect is that of a time controlled implant.

Increasing in general complexity from simple diffusive implants are time controlled implants. At their simplest, time controlled implants are composed of a number of reservoirs sealed with caps that erode away over time to release their stored medication. By varying the thickness of the caps, the release of the medication will be spaced out in time accordingly. However, since making different thicknesses of caps is difficult on the microscale, an alternative is to make caps with equal thickness, while using an electrochemical erosion process, with each cap linked to a common source. At any given time the circuitry will only connect to one cap, electrochemically breaking it down until the seal ruptures, releasing the medication in the reservoir. The ruptured seal also acts as a circuit breaker, transferring the current causing the erosion to the next cap in the sequence. Since this process is very easy to control, it allows for the delivery of the medication to be spaced out in time with a high degree of control. These sorts of anode-cathode microchip reservoirs have been worked on extensively by Santini et al. [14,15]. Of interest with this sort of design is that while applying a current to the eroding anodes can be done at a constant rate, the microchip design can package a control clock into the device to space out the release pattern in time further, or to only run the current upon user activation, or in response to specific physiological changes that it senses. Thus, for a number of microfabricated devices they can be connected to different triggering mechanisms and be used as time controlled, user activated, or condition sensing devices. Some devices work best with certain activation methods rather than others, depending particularly on what drugs they are releasing. A further example of implantable microchip-based drug delivery technology was developed by Debiotech (Figure 8b). The MIP micropump represents the main component of the implant and it is obtained by standard silicon micromachining technology [136]. The pump is piezo-actuated and it consists of two plates in silicon containing the pump structures, two glass layers, and a piezoelectric ceramic disc which actuates the titanium fluid connectors. This fundamental technology can be incorporated in a more complex device to obtain a programmable implant for drug infusion.

As an example of a user controlled medication releasing implant based on microfabrication technology, there is a design for a refillable microfabricated drug delivery system for the treatment of ocular diseases [137]. Using silicon and acrylic masters, the silicon being etched to specification using microfabrication techniques, a PDMS device was constructed using molding and bonding together multiple layers. Consisting of a reservoir and cannula with support structures to be secured to the sclera of the eye, the device allows for medication to be delivered into the interior of the eye. Release is activated by applying mechanical pressure to the reservoir to force medication through the cannula, and the device can be refilled by using a syringe and needle to pierce the reservoir. The device is robust enough that with careful needle selection and a steady hand during the refilling process allows for an expected 24 refills, with an expected refill frequency of 3 months at one delivery per day, meaning that the device will have a lifetime of approximately 6 years. This demonstrates the utility of a simple device in that one surgery to implant the device and insert the cannula saves the patient from over 2000 injections into the eye, or damage to the eyes from a lack of treatment, or unwanted systemic side effects from having to use a route that would deliver the medication to the entire body.

Another example of a user controlled implant is the MEMS device developed for rapid delivery in ambulatory or out-patient care patients [138]. Designed to deliver a small number of bolus shots via film boiling of material actuated by resistive heating elements contained within a microcapsule that can be inserted under the skin, this device varies wildly from the idea of an implant for long term administration of pharmaceuticals. Instead, its purpose is either ambulatory or in-patient care, particularly of patients who may experience emergency medical events. By installing the implant, the patient can have access to an on-demand shot of medication ready to be delivered at any time during care. While requiring removal after use, either for replacement or because the treatment regime concluded, such implant can provide immediate delivery of medication—potentially directly to the required organ—at the press of a button. This adds the speed and convenience of drug delivery from an IV line as would be available with in-patient care, but not requiring the IV stand that would be impractical for use in out-patient care.

One major use for microfabrication in producing improved drug releasing implants is the possibility of immunoisolation of implanted cells via nanoporous membranes [139]. The system works by using microfabrication to create a silicon membrane with pores small enough that large molecules like antibodies and cytokines cannot pass through to cause cell death, while small molecules like glucose, oxygen, carbon dioxide, and insulin can enter. This allows transplanted cells to survive and produce insulin for a patient in a manner as close to natural function as possible, without the normal difficulties of transplanting foreign cells into the body. A similar type of implant in commercial development is the βAir Device, featuring an alginate and polytetrafluoroethylene (PTFE) immunoisolation membrane [140]. It is currently undergoing human trials [141] and has shown in the preclinical trial stage the capacity for xenotransplantation of rat islet cells into diabetic pigs [142]. While this device allows users to not have to inject insulin or anti-rejection drugs, it does require daily injections of oxygen to a reservoir to maintain the health of the cells.

### 3.3. Oral Delivery and Detection

#### 3.3.1. Background

Environment-sensing microencapsulation systems offer a unique opportunity to carry functional ingredients in their core and to release them in response to environmental stimuli (temperature, pH, osmolarity, etc.) for desired effects. In particular, pH-responsive encapsulation systems which can load/release functional substances by sensing environmental pH change have received much attention from both academia and industry due to their diverse applications in food industry (nutrient delivery [143], packaging [144]), depollution (adsorbent of contaminants or carrier of chemical/biological reactants) [145], biochemical technology (protein separation [146] and reaction catalyst [147]), cosmetic (emulsifier, pH regulator, preservative, antiaging actives, skin whitening, and emollient) [148,149], wound healing [150], and disease prediction/treatment (animal/human oral pharmaceuticals/vaccines [151], insulin [152], anticancer therapy [153], and imaging [154]). Despite these efforts, current encapsulation systems are limited to small molecules due to low encapsulation efficiency, poor stability, and inefficient release profile. Consequently, efforts have been focused to develop a universally applicable microencapsulation platform with tailored pH responsiveness.

**Figure 9:**
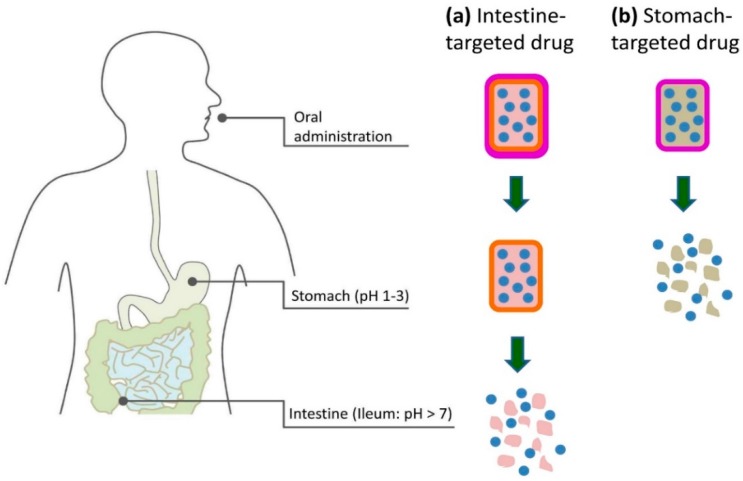
General scheme for the deployment of pH sensitive medication to the gastrointestinal tract ((**a**) Intestine-targeted drug; (**b**) Stomach-targeted drug).

The defining condition for the oral route is the low pH, typically about 2.0, in the stomach and the neutral pH of about 7.0 in the intestine [155]. The acidity of the stomach is physiologically important for digesting and serves as a barrier for infection, but it also makes delivery of medication difficult as the low pH disrupts the active compounds. This is particularly a problem for intestine-targeted biopharmaceuticals, as the digestion of the stomach is primarily to denature proteins to make them easier for enzymes in the small intestine to dismantle into their component amino acids for absorption. As such, the only way for pH vulnerable compounds that are absorbed in the intestines to cross the barrier posed by the stomach, is to be encapsulated within a compound that will not be degraded by the low pH environment [1]. Ideally, such encapsulation would remain stable at acidic environments and then undergo some form of change, such as dissolution or swelling, with varying pH around 7.0 to release the active ingredients only when exposed to the targeted pH in the intestine (Figure 9a). On the other hand, stomach-targeted pharmaceuticals are required to satisfy both pH stability in neutral environment and rapid release in acidic environment [156] (Figure 9b). Therefore, the key structural feature of oral delivery system is the presence of pH-responsiveness that can sense and adapt to environmental changes. This means that functional substances encapsulated in these delivery systems would be protected in unfavorable pH conditions, and would be released in the favorable pH environment.

To implement aforementioned objectives, significant efforts have been focused to develop a platform using pH-responsive polymers (i.e., anionic and cationic polymers). A pH-responsive swelling/shrinkage behavior of encapsulation systems can be accounted for by the conformation change of polymer chains at different pH (i.e., extended or coiled). Ionization of weak acidic (i.e., carboxylic or sulfonic acid) and basic (i.e., amine) functional groups in the backbone of the polymer plays a key role in the swelling of encapsulation system made of polyacidic and polybasic polymers at high pH and low pH, respectively [157]. The opposite holds: deionization of the functional groups will induce shrinkage of polymers by forming interpolymer complex. Hence, anionic copolymers (e.g., methacrylic acid-methyl methacrylate copolymer) or cationic copolymers (e.g., poly(butyl methacrylate-*co*-(2-dimethylaminoethyl) methacrylate-*co*-methyl methacrylate) have been widely investigated as the core parts of intestine-targeted and stomach-targeted drug delivery systems, respectively [158,159]. Therefore, design of new architecture with optimal drug formulation can promise successful development of target-specific, pH-sensing microencapsulation systems.

#### 3.3.2. Status

At the time of publication, there are no commercial products on the market featuring microfabrication-based oral drug delivery systems. The oral route does however remain of crucial importance because of the ease of administration and the possibility of engaging with specific features within the gastrointestinal (GI) tract, namely pH variations. Moreover, there are useful immunological components of the GI tract that make it of interest for engaging the mucosal and humoral immune system [160]. In particular, the Peyer’s Patches of the ileum, the last part of the small intestine, are organized nodules of lymphoid tissue and thus present a direct route to the humoral immune system. For certain medications, particularly vaccines, being able to deliver directly to these regions would be of significant benefit over other methods.

The use of various techniques to fabricate micro or nano particles that respond to pH is not just useful for simple delivery, but also for more advanced concepts. By using a polymer that swells in response to pH change, a hollow particle that opens in the small intestine could be generated. Going beyond that, by functionalizing the surface, or possibly only one side of the surface, of the particle, it could be made to adhere to the lining of the intestine during delivery to increase the amount of medication taken up by the body, rather than passing through the GI tract without being absorbed. Such functionalization could allow adherence to immunological features such as Peyer’s Patches, with direct application of pharmaceuticals to the lymphatic system and the immune responses contained within. This in turn could allow for very low concentrations of high potency medications or vaccines to achieve dose sparing effects.

While not medication, there is already a major intersection between microfabrication and medicine involving the oral route in the form of capsule endoscopy [161,162]. Microfabrication allows for the production of electronics small enough for a digital camera complete with flash and wireless transmitter to fit into a capsule the size of a pill that can be swallowed, and survive transit through the GI tract. Such capacity to hold microelectronics in a compact package suggests the possibility of combining together a sensor to detect prevailing conditions and a drug reservoir that could be opened or closed on command [123]. This is conceptually similar to some of the more advanced forms of drug delivery implants proposed, whereby internal sensors can release medication from a reservoir in response to external conditions. The primary difference is that in the case of the oral delivery route residency time within the body would be shorter compared to that of an implant.

**Figure 10:**
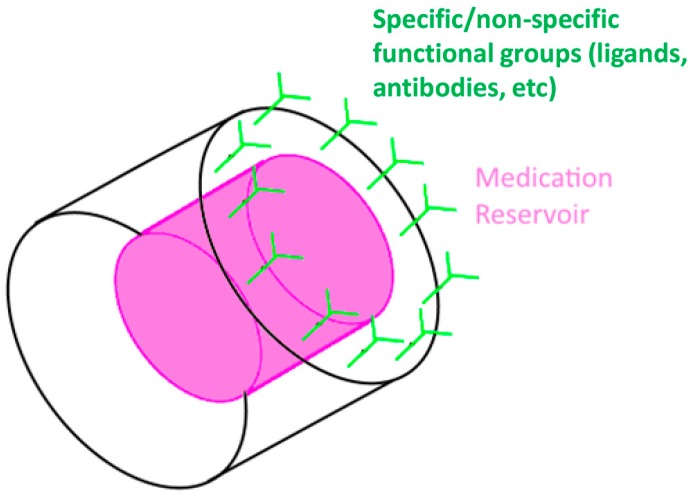
Schematic of a possible way to produce a drug delivery system with site specific/non-specific functional groups on one side, so that the pharmaceuticals stored within will diffuse out directly upon the desired location.

To increase the efficiency of delivery, micro or nanoparticles can have modified surfaces to increase attachment to the intestinal walls, residency time within the GI tract, transport across intestinal mucosa, and uptake by intestinal cells. To improve target specificity, physicochemical/biochemical properties of microencapsulation systems can be easily varied by adding another material to the O/W emulsion used to make microcapsules. For example, to improve pharmaceutical transport across epithelial cells, the surface can be modified to enhance adhesion [163]. Non-specific approach includes coating/grafting with mucoadhesive polymers (PVA, chitosan) [164,165]. Specific approach includes targeting enterocytes by coupling with LTB/TL lectin and glucomannan [166,167], or by targeting M cells in the gut-associated lymphoid tissue by coupling with UEA-1/WGA lectin and RGD peptides [168,169,170,171]. Although these approaches were utilized for enhanced uptake of drug-loaded nanoparticles, they can be extended to increase association of microfabricated drug delivery systems with intestinal cells, allowing release of ingredients at the site of absorption. Thus, concentrated ingredients with increased residence time can facilitate uptake by intestinal cells. While simple microparticles are likely to be spheroidal, by using microfabrication asymmetrical particles can by synthesized [172,173] and the specificity and effectiveness of the oral route can be further refined. For example, ligand agents can be applied to only one side of the particle that releases the drug, and this face will have a flat surface to attach efficiently to the intestine (Figure 10). Ideally, these structures will only adhere to certain parts of the intestine, and then release directly at these sites, to maximize the amount of medication delivered directly to the necessary locations. Furthermore, such capsules are likely to be encapsulated in a pH-sensitive polymer to protect their surface chemistry from degradation in gastric conditions, with possibly another sealing agent over the reservoir that can be easily eroded in enteric conditions, such as gelatin [174].

In addition to ligands, there are other compounds that could be attached at the same location to further enhance effects, such as chemicals to increase the permeability of endothelial cells and mucus layers to penetration by the active agents. Combining such agents with specific or non-specific functional groups can however run into complications as the agents could compete with each other for occupation of the surface, producing suboptimal results. Fortunately, the same microfabrication techniques that allow for the fine structure control required to produce such structures allows for the selective patterning of regions to have different surface modifications [31], such as checkboard patterns of different exposed materials with different chemical affinities.

It should be noted that this general design of an encapsulated set of drugs combined with functionalized system extends to even smaller structures. If made as extremely fine microparticles or as nanoparticles, such structures could be introduced into the bloodstream or the lymphatic system to circulate in the body until encountering the target cells [175]. This is a particularly promising idea for the targeted delivery of chemotherapy drugs to cancer cells, in that it will ensure that cytotoxic chemicals are delivered only to cancer cells, leaving healthy cells unaffected [176]. This has the advantage of reducing the adverse side effects on patients, and can allow for the medication to be circulated generally within the body to also affect any metastatic cancer cells that have migrated away from the primary tumor.

It is to be emphasized that for oral delivery methods the greatest strength microfabrication techniques can bring is that the fine size control allows for a greater density of desirable features, which can also be stacked hierarchically. An example of a system that would involve many of the discussed techniques would be a pill that can be swallowed, containing polymer microparticles that can survive at low pH, but dissolve under neutral conditions, releasing functional pharmaceuticals. 

### 3.4. Vaccine Delivery Systems

#### 3.4.1. Background

Vaccine delivery systems present notable differences from conventional drug delivery systems, therefore possessing unique technical requirements. A prominent aspect of vaccination is to elicit disease-specific protective immune response as well as memory response [177]. For this purpose, it may contain adjuvants, along with the antigen, which enhance the immunogenicity of the vaccine. Moreover, the delivery system should successfully penetrate barriers of the body to effectively allow the antigens to stimulate systemic or mucosal immune responses.

Vaccine design has not generally deviated from the original principles first developed by Louis Pasteur: isolate, inactivate, and inject the causative organism. Improvements in the technology of vaccination has primarily come from improvements in the first two steps, with new techniques to find and isolate pathogenic organisms, and new ways to inactivate the pathogens for presentation to the immune system [178]. The methods of delivering the vaccine to the patient have not significantly changed, despite the fact that intramuscular injection is often not the most favorable route, from a logistical or immunogenic perspective.

Fundamentally, vaccines face different development pressures from other pharmaceuticals. Most medicines prefer to avoid an immune response from the body, since it typically degrades and/or eliminates the compounds, reducing their effects. In contrast, the purpose of vaccines is to elicit an immune response so as to exercise the adaptive immune system, and thus develop immunological memory. Ideally, this mechanism should not trigger an excessive reaction of the innate immune system, and for the purposes of safety the active forms of the pathogen are minimized. 

Because the purpose of a vaccine is to engage the immune system, an important part of the design process is the inclusion of adjuvants in the formulation. Adjuvants are ingredients of the vaccine that serve to enhance the function of the primary component. They can allow a lower dosage of antigen presenting substance to be administered to the patient, thus increasing the safety of the vaccine while not decreasing its efficacy.

For human and veterinary vaccines, aluminum compounds are among the most common forms of adjuvant, most typically aluminum oxide and aluminum phosphate [179], both of which are mostly insoluble under physiological conditions [180]. Particularly, human vaccine formulations that contain adjuvants are *Haemophilus influenzae* type b, hepatitis A and B, human papillomavirus, meningococcus, pneumococcus, tetanus toxoid, and inactivated influenza H1N1 and H5N1 (which differently from other vaccines uses oil-in-water emulsions-based adjuvants ASO3 and AFO3) [181]. While the precise mechanisms by which aluminum compounds promote improved immunogenic responses are not entirely understood, it is known that there is a relationship between the amount of antigen adsorbed on the surface of the aluminum compounds, along with the dose of adjuvants, and the vaccine immunogenicity [179]. The two mechanisms believed to contribute to the adjuvant effect are depot formation and inflammatory response [179]. The depot formation theory is the concept that, since the aluminum compounds are insoluble under physiological conditions, there will be micro and nanoscopic clumps of aluminum left at the injection site for weeks or months after the injection, releasing vaccine particles into the body over an extended period. This means that more of the antigens will be directly engaged by the immune system instead of being removed, promoting the development of immune memory. The inflammatory response theory has to do with the fact that aluminum compounds naturally provoke the innate immune system. Thus, aluminum micro and nanoparticles loaded with adsorbed vaccine compounds will be attacked by macrophages seeking to eliminate them from the body. This helps ensure that the immune system is more largely exposed to the antigens.

The exact mechanism of how aluminum improves the immunogenic activity is primarily of importance in the search for other adjuvants. One particular point is that vaccines with combinations of antigens have been shown to have stronger immunogenic effect than each antigen independently, such as combining together cholera toxoid with diphtheria toxoid [182] or influenza subunit vaccine [183].

Ideally, a vaccine will induce strong humoral and cell-mediated immune responses, which provide protection against specific viral infections. Also important to vaccine design is how the site and route of delivery will influence immune response. Because immune systems involve different reaction mechanisms against vaccine, the selection of administration routes can determine types of immune response elicited and their quality. For example, intramuscular (IM) injection does not engage with the mucosal immune system in contrast to activation of both mucosal and systemic immune response by intranasal (IN) and oral vaccinations [184]. Unfortunately, in the case of vaccine delivery, the front lines of the immune system are too effective, with physical barriers such as the stratum corneum of the skin [185], or chemical barriers such as the low pH conditions of the gastric juices in the stomach [186]. To obtain the best performance from vaccines, the delivery method should bypass the physical and chemical barriers, but engage immediately with the cellular components of the immune system, so that vaccine particles can maximize long lasting protective immunity.

#### 3.4.2. Status

The U.S. Food and Drug Administration’s list of approved vaccines includes 80 approved products [187]. The great majority of these products are administered via intramuscular injection, with a small number being delivered orally, via intranasal spray, or percutaneous delivery. It can thus be safely said that the majority of vaccines delivered worldwide are done so via intramuscular injection using hypodermic needles. As a consequence, current vaccine delivery has several requirements that increase expense and decrease coverage.

The first aspect is the cold chain. In order to allow for safe usage, the vaccines must be kept under refrigerated conditions up to the point of delivery. As a result, from the point of production to the point of delivery refrigerated transport and storage is needed, which are expensive [188]. In developing nations—which incidentally also have a tendency to have hot climates—this serves as an infrastructural impediment to safe and widespread delivery of vaccines [189]. Vaccines that can be stored at room temperature or even at elevated temperatures and remain safe and effective are thus preferable to ones that require refrigeration, as it allows for less expensive and more widespread delivery globally.

Second is that intramuscular injection requires the use of skilled healthcare workers to give the injections safely and properly. This imposes further costs and logistical issues [190]. The third issue is that intramuscular injection involves the generation of biohazardous sharp waste [191], which imposes disposal costs and creates risks for the transmission of blood borne diseases, if not handled appropriately. Finally, intramuscular injection is not necessarily the best route for the development of robust, long lasting immune memory [184].

However, because of the large components being delivered by vaccines, the smallest units being complex proteins and the largest being whole bacteria, intramuscular injection is often the only route whereby a sufficiently large number of antigen compounds can be delivered to the body properly. As such, intramuscular injection is the gold standard against which all new methods must be compared. If a new method is not at least comparable to intramuscular injection in terms of immunogenic efficacy, it is unlikely to meet with approval.

Vaccine delivery via transdermal microneedles is an exceedingly promising method of delivery in that it could decrease costs and increase impact by not requiring a skilled healthcare worker to deliver the vaccine intramuscularly and increase vaccination rates among needle-phobic individuals, a known barrier to full immunization among adults and children [192]. Additionally, the vaccines can be contained within the needles as dry compounds, which reduces or even eliminates the need for maintaining a cold chain [189]. Ensuring efficacy and safety does however mean that these applications are still in the testing stage.

**Figure 11:**
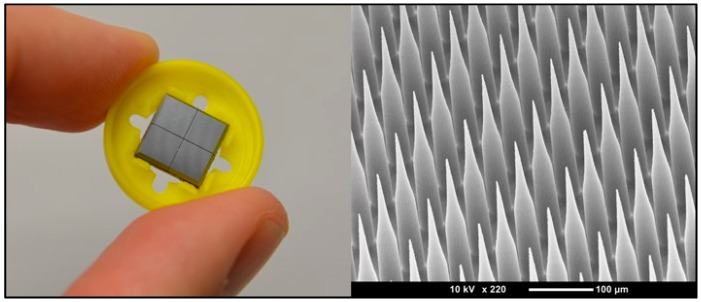
Nanopatch technology for vaccine delivery (image provided courtesy of Vaxxas Inc., Cambridge, MA, USA).

Influenza subunit vaccines coated on microneedle patches have been shown to perform at an equal level as intramuscular injection in stimulating immune responses in guinea pigs [193]. Use of excipients like trehalose can stabilize inactivated virus influenza vaccines on coated metal microneedles [194]. Comparisons between influenza vaccine delivered to mice via microneedles and intramuscularly showed that the microneedles produced antibody titers with the same speed, strength, and longevity of the traditional method [195]. The availability of these sorts of patches is particularly important as the possibility of self-administration should increase yearly vaccination rates against influenza significantly [196], with the combination of cheaper patches and higher vaccination rates having major potential economic benefits [197]. Metal microneedle patches coated with inactivated rotavirus vaccine were found to induce an immune response in BALB/c mice greater than an intramuscular injection with ten times the dose of vaccine, indicating a possible dose sparing effect [198]. Such effects, including an adjuvant sparing effect, have also been observed when comparing intramuscular delivery to microneedle delivery of influenza vaccine in C57BL/6 mice using Nanopatch technology [119] (Figure 11). Polio vaccine is currently administered orally because it can be delivered widely around the world in that form without need for the infrastructural elements of injected vaccines. Unfortunately, it also carries the risk of reverting to a virulent type, and as such there is now pressure to move to an inactivated type polio vaccine. It has however been shown that microneedle patches can deliver inactivated polio vaccine and generate immunogenic results in rhesus macaques [199]. For measles vaccination, immunization via microneedle patches has been demonstrated to work in cotton rats [200] and rhesus macaques [201].

Microneedle patches represent a successful microfabricated drug delivery system. Microneedles are fabricated to have several hundred micrometers, thus can deliver vaccines near epidermis/dermis layer. This indicates that microfabricated needles enable precise transdermal vaccination in a reliable manner which was not possible by conventional hypodermic needles [202]. In addition, microneedle vaccination stimulates antigen presenting cells, such as Langerhans and dermal dendritic cells in the skin for immune response [203]. Thus, the meaning of microneedle compared to hypodermic needle can be extended to indicate higher immunity and/or different immune mechanism.

In contrast to transdermal vaccination using microneedles, no significant progress has been made in developing oral vaccines due to significant technical challenges: (i) protection of vaccine in the stomach and (ii) efficient release/uptake in the intestine. This is related to two seemingly contradictory requirements in the encapsulation system: structural stability of encapsulated ingredients and their efficient release. As a result, conventional encapsulation systems failed to meet one or both of the above requirements. Importantly, an increase in the size of ingredients tends to magnify the problems associated with encapsulation/release. Hence, conventional encapsulation systems cannot meet the demands of high-precision performance, mainly because of their architectural limits.

## 4. Prospects

MEMS have found broad application in health and pharmaceutical sciences in recent years, with drug delivery being an exceedingly explored field. Drug delivery has drawn multiple advantages from employing microfabrication technologies, such as high selectivity of target site [5], size control at the micro and nano scale [11] and potential for multifunctional platforms [10]. The use of processes developed for the semiconductor industry and introduction of new techniques such as soft lithography and micromolding allowed the development of advanced drug delivery systems through the oral and transdermal route as well as drug eluting implants.

Microfabrication offers numerous features that have the potential to overcome current challenges in drug delivery systems. A first compelling advantage is the precise fabrication of complex architectures having tight tolerances [12]. Unlike conventional drug delivery systems based on solid or hollow particles, gel, and vesicles, easy production of complex parts with diverse functionality is one of the significant advantages microfabrication offers over conventional chemical synthetic methods. This allows researchers to take advantage of controlling architectures in addition to formulation for improved drug encapsulation/release, better protection against harsh environments, and environment-responsive controlled transport. As an instance, in the case of conventional nano/micro polymeric particles, ingredient encapsulation occurs during the particle formation process [204]. Thus, the harsh chemical/thermal conditions required for particle fabrication result in a loss of drug functional activity. Additionally, another common process that lowers drug activity is high concentration of detergents [205], which are used to facilitate drug encapsulation. In contrast to these conventional methods, stress applied to the active ingredients can be reduced by loading them into a premade or intermediate microfabricated architecture with pore-like opening. Moreover, the absence of organic solvent during drug encapsulation into the microfabricated system can also contribute to the increase of functional efficacy of drugs [206]. For instance, unlike conventional drug encapsulation process of mixing delivery vehicles with drug suspension, micromolding-based drug casting process can be useful in loading highly concentrated drug formulations onto microfabricated structures by simply applying vacuum. With this method, drug loss can be minimized due to the benefits of planar mold architecture, as remaining drug after encapsulation can be easily recovered by pipetting. Therefore, microfabrication of precise, complex architectures promises to produce multifunctional, highly loading-efficient drug delivery systems.

Additionally, microfabricated drug delivery systems can guarantee control over drug release profiles [12], achieving constant doses and reduced toxicity due to overdose. In particular, this can help to cope with drugs with a narrow therapeutic index, such as carbamazepine, digoxin, phenytoin, theophylline and warfarin [207]. Inconsistent fluctuation in drug concentration has been recognized as a highly significant challenge of conventional oral drug formulations for sustained and safe therapeutic effects of drugs. In fact, slight changes in concentration of narrow therapeutic index drugs can easily cause either toxicity or ineffectiveness due to the limited range in which the ideal dose, with high therapeutic effects and no severe adverse effects, lies [208]. The microfabrication process can be designed to have multiple-functional layers to exhibit environment-specific, time-dependent responses of oral formulations in a controlled manner. Similarly, proper selection of materials and design of layered structure of the system can be used to increase or decrease the absorption window of oral drug formulations. Lastly, architectures with multiple drug loading divisions can be produced to develop multi-target drugs and sequential drug releasing systems. 

A further challenge of drug delivery systems is maintaining stability, not only during encapsulation, but also during storage and application. Especially, storage stability is recognized as one of the greatest challenges in vaccine technology. Optimal efficacy of vaccine is determined by formulations and management conditions. However, the current liquid form of vaccines has several disadvantages, including short-term stability and need of proper infrastructure (i.e., electrical cold chain systems for transport, shipping, and storage) [188]. Consequently, the vaccine price raises and the hurdle when stockpiling increases. The limited shelf-life and strict cold chain requirements can be attributed to hydrolytic degradation and hypersensitivity to temperature change. Stability profile exhibit a wide range of variation in lifespan among vaccines; i.e., 6 months for polio vaccines (mono, bi, and trivalent), 6 months for pandemic influenza vaccines (H1N1/H5N1), and 6–12 months for seasonal influenza vaccines (split/whole) in their shelf-life at 2–8 °C [181]. Furthermore, vaccines are damaged by accidental freezing in the cold chain as well as by temperature increase, thereby resulting in the decrease of their shelf-life to 2–4 weeks at 25 °C, and one day at 37 °C. Such an intrinsic vulnerability of temperature-dependent vaccine stability highlights the importance of temperature controls at all levels, from vaccine manufacturer to health clinic. However, poor vaccine supply chains in low- and middle-income countries have been acknowledged as the major obstacle to achieving global vaccination [189]. Thus, development of solid vaccine featuring characteristics such as cold chain-independence and thermal and long-term stability, is extremely desirable. It is important to note that shelf-life of vaccine can be improved through thermodynamic parameters, by controlling the size/geometry of vaccine-embedded architectures, and kinetic parameters, by optimizing solid vaccine formulations. It should be emphasized that development of long-term stable vaccine formulation remains the major unsolved question for universal application of microneedle technology. Therefore, future research in microfabricated vaccine delivery systems will be directed to develop long-term stable, solid vaccines due to their impact on global vaccination strategy.

The salient features of MEMS include rapid production capability, consistent end-product quality, bulk manufacturability, and flexibility in material selection and design. These unique abilities offered by MEMS technology represent a promising tool to complement conventional synthetic drug delivery systems. Ultimately, microfabricated drug delivery systems will give impetus to current drug delivery/vaccination technology by uncovering an alternative set of solutions.

## 5. Conclusions

Current technical problems in health, prominently drug delivery, have been increasingly addressed by microfabrication. Researchers have found that microfabrication technologies display a broad panel of appealing features for the design and production of drug delivery systems. Therefore, due to incomparable advantages over other fabrication technologies, microfabrication is envisioned to provide continuous novel approaches to drug delivery and health.
